# Tailoring optical complex field with spiral blade plasmonic vortex lens

**DOI:** 10.1038/srep13732

**Published:** 2015-09-03

**Authors:** Guanghao Rui, Qiwen Zhan, Yiping Cui

**Affiliations:** 1 Advanced Photonics Center, Southeast University, Nanjing 210096, China; 2 Electro-Optics Program, University of Dayton, 300 College Park, Dayton, OH 45469, USA

## Abstract

Optical complex fields have attracted increasing interests because of the novel effects and phenomena arising from the spatially inhomogeneous state of polarizations and optical singularities of the light beam. In this work, we propose a spiral blade plasmonic vortex lens (SBPVL) that offers unique opportunities to manipulate these novel fields. The strong interaction between the SBPVL and the optical complex fields enable the synthesis of highly tunable plasmonic vortex. Through theoretical derivations and numerical simulations we demonstrated that the characteristics of the plasmonic vortex are determined by the angular momentum (AM) of the light, and the geometrical topological charge of the SBPVL, which is govern by the nonlinear superposition of the pitch and the number of blade element. In addition, it is also shown that by adjusting the geometric parameters, SBPVL can be utilized to focus and manipulate optical complex field with fractional AM. This miniature plasmonic device may find potential applications in optical trapping, optical data storage and many other related fields.

As one of the important properties of light, the optical angular momentum (AM) describes the amount of dynamical rotation and can be divided into spin angular momentum (SAM) and orbital angular momentum (OAM)^1^. The SAM is associated with the intrinsic polarization helicity, where *σ*_±_ = ±1 correspond to the right and left-handed circular polarizations (RHC and LHC), respectively. The OAM arises from the spatial structure of the optical field and has a value of *lħ* per photon (*l* can take any integer value). In the past decade, optical complex fields with unconventional spatial distributions in terms of amplitude, polarization and phase are rapidly becoming a current trend due to the possibility of exploring the fundamental physics with numerous potential applications including optical tweezers, information storage, processing and transportation, microscopic and nanoscopic imaging, remote sensing, materials micromachining and processing, etc^2^,^3^,^4^,^5^,^6^. Among those optical complex fields, vortex beam is one specific case that is characterized by a helical wavefront along with an optical singularity in the center. As a well-known class of optical scalar vortex beam, Laguerre-Gauss mode LG_*pl*_ has azimuthal symmetric intensity pattern and a helical phase structure described by *e*^*jlϕ*^, for which each photon carries OAM of *lћ*. Optical vortex beams have been exploited in various forms for many applications ranging from optical spanners, and twisted light for telecommunications, to faster data manipulation in quantum computing^7^.

Surface plasmon polaritons (SPPs) are free electron oscillations near metal/dielectric interface due to the interactions between incident photons and conduction electrons of the metal. The resonant interaction causes many unique properties of SPPs, such as short effective wavelength, high spatial confinement and strong field enhancement^8^. One of the most attractive aspects of the SPPs in the field of optics is its capability of concentrating and channeling light with subwavelength structures, enabling miniaturized photonic circuits with dimensions much smaller than those are currently available. When light pass through a metallic structure with size comparable to the wavelength of the light, the coupling of light to SPPs leads to plenty of unusual optical behaviors such as extraordinary optical transmission^9^,^10^, beaming^11^,^12^, plasmonic focusing^13^,^14^, and superresolution^15^. Besides, the strong confinement of light provided by the subwavelength metallic structure with anisotropic inhomogeneous boundaries gives rise to strongly non-paraxial regime that has large intensity gradients on the wavelength scale, in which the spin and orbital angular momentums of light are no longer independent physical quantities but are tightly coupled^16^. This change depends on the polarization of the incident light and the topology of the medium, the relationship of which can be described by the concept of geometrical phase (or Pancharatnam-Berry phase)^17^. Recently, this phenomenon has enabled geometric phase devices that can control the wavefront of the output beam according to the polarization of the illumination. The geometric phase can be created with coupled isotropic scatters^18^, or using anisotropic subwavelength scatters with spatially varying orientations^19^,^20^. The concept of the spin-orbit interaction has been widely used in considerable number of studies that are devoted to design nano-metallic structures capable of receiving and distinguishing the optical complex field with high efficiency and fidelity in convenient way. For example, according to the polarization mode match theory, the receiving efficiencies of the optical beams with radial and circular polarizations can be maximized with bull’s eye and spiral Archimedes’ plasmonic lens, respectively^13^,^21^. Besides, these plasmonic structures also can be used to detect AM of light or generate SPPs vortices^22^,^23^,^24^.

In this work, we proposed a spiral blade plasmonic vortex lens (SBPVL) that is suitable for tailoring the optical complex field into plasmonic vortex. The overall effect arising from the structures of both the optical field and the SBPVL on the characteristics of the synthesized SPPs vortex is also studied. Both the theoretical calculation and numerical simulation results demonstrate that the OAM per photon of the SPPs vortex inherits the AM of the light beam and the twisted metal/dielectric interface, enabling a method to generate plasmonic vortex with almost arbitrary integer topological charge (TC). In addition, it is found that the geometrical TC of the SBPVL is a nonlinear superposition of the pitch and the number of blade element. Furthermore, the optical AM conservation law during the process of SPPs vortex generation is validated as an evidence of the spin-orbit interaction in this plasmonic system.

## Results

### Theoretical expression of the plasmonic field

**Figure 1:**
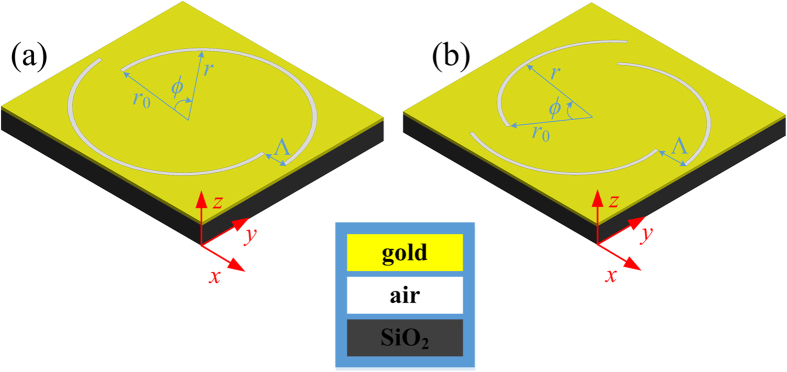
Diagram of the SBPVL and the coordinate setup for the plasmonic field calculation. The SBPVL consists of *m*-curved slot with the pitch of *n* in the units of *λ*_*spp*_. (**a**) (*m, n*) = (2, 2). (**b**) (*m, n*) = (3, 3).

**Figure 2:**
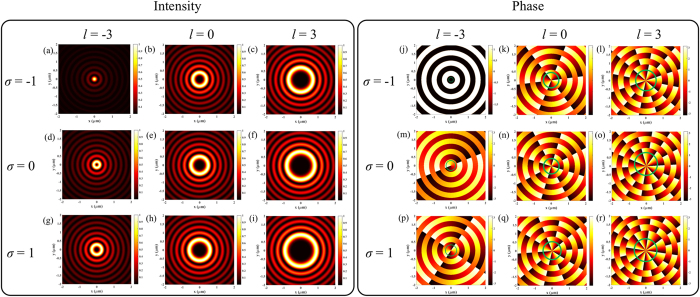
Analytical calculation results for the SBPVL with (*m, n*) = (2, 2) and illumination with different AMs. (**a–i**) Intensity and (**j–r**) phase distributions. The top, middle and bottom rows are corresponding to LHC (*σ* = −1), radial (*σ* = 0) and RHC (*σ* = 1) polarizations, respectively. The TC of illumination varies from –3 to 3 with increment of 3 from the left to the right column in each panel.

The proposed SBPVL design and the coordinates for calculation are illustrated in Fig. 1. The SBPVL is consisted of several curved slots etched into a thin gold film. In its local cylindrical coordinates, the SBPVL can be described as:
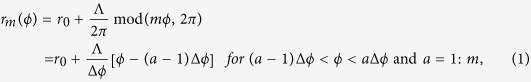
where *m* denotes the number of blade element, *r*_0_ is a constant indicating the distance from the geometrical center to the innermost edge of the SBPVL, mod(*mϕ*,2π) represents the reminder of the division of *mϕ* by 2*π*, and Δ*ϕ* = 2*π/m*. The pitch of the SBPVL (Λ) is defined as Λ = *nλ*_*spp*_, where *n* is an integer and *λ*_*spp*_ is the wavelength of SPPs propagating on the gold/air interface. Considering a normally incident optical complex field with OAM of *l* and SAM of *σ* propagating towards *z* direction, it can be expressed in the cylindrical coordinates as:
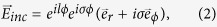
where *e*^*iσϕ*^ is the Berry phase and the *e*^*ilϕ*^ is the phase due to the OAM. The incident light is coupled into SPPs by the metallic edge of the slot opening. Note that only the radial component of the optical field can excite SPPs. The plasmonic field at an observation point (*R, θ*) near the origin due to the excitation along an incremental length of the slot is given by:
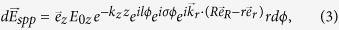
where *E*_0*z*_ is a constant related to the coupling efficiency of the illumination, 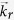 is the transverse wave vector of the SPPs that propagate from the arm to the geometrical center, *k*_*z*_ is the longitudinal wave vector of the SPPs that propagate normal to the gold surface. The subwavelength slot opening can be regarded as an array of secondary sources. Consequently, the electric field at an observation point can be expressed as:
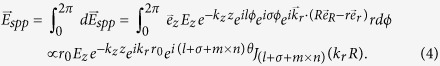


Please refer to the Methods for the detailed derivation. From equation (4) one can see that an optical complex field with (*σ, l*) is tailored by a SBPVL with (*m, n*) into an evanescent Bessel vortex beam with TC of (*σ* + *l* + *m* × *n*). The AM information of the incident light is conserved and imprinted into the OAM of the plasmonic field, therefore the properties of the illumination plays a vital role in determining the characteristics of the synthesized vortex field. With the analytical expression given in equation (4), the contribution of the incident AM to the intensity and phase distributions of the plasmonic field are studied and presented in Fig. 2. Please note that a SBPVL with (*m, n*) = (2, 2) corresponding to the structure shown in Fig. 1(a) is used and kept unchanged. In this case, different polarizations of light are studied, including LHC (*σ* = −1), radial polarization (*σ* = 0) and RHC (*σ* = 1), which are corresponding to the top, middle and bottom rows of Fig. 2, respectively. Radially polarized beam is one special case of cylindrical vector beam with its local polarization aligned in the radial directions^25^. Besides polarization, the spatial structure of light is also taken into consideration. From the left to the right column of both left and right panels of Fig. 2, the TC varies from −3 to 3 with increment of 3. It is known that the circular polarization can be decomposed into the combination of radial and azimuthal components along with a geometric phase term^25^, while the azimuthal component cannot be coupled to SPPs since it is TE polarized with respect to the curved slot. In this sense the radial polarization has similar effects with circular polarization (no matter what handedness is) in terms of SPPs excitation with respect to the SBPVL. However, as shown in Fig. 2, for arbitrarily specific TC the intensity and phase distributions are completely different for light with different spins. These phenomena can be accounted by the Berry phase that purely arises from the mathematical decomposition. Besides, different TCs also lead to different plasmonic field for incident light with fixed SAM. In addition, it should be noted that the intensity distributions are the same for configurations with the identical total value of (*σ* + *l* + *m* × *n*), that is, the generation of SPPs vortices with the same TC. In this closed physical system, there is a selection rule which associates with the AM that can be attributed to the conservation of the total AM. It can be expressed as:
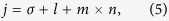


where *j* denotes the TC of the plasmonic field. For example, when the AM of the light can be compensated by the SBPVL (*σ* + *l* = −*m* × *n*), the phase distribution of the plasmonic field near the geometrical center of the SBPVL is uniform, namely the TC is 0, leading to a focused solid spot. In the other cases, a doughnut shaped field with a dark center is obtained, indicating the non-zero OAM carried by the SPPs. Please note that the TC of the plasmonic field can be visualized by inspecting the phase along closed contours within the main lobes.

**Figure 3:**
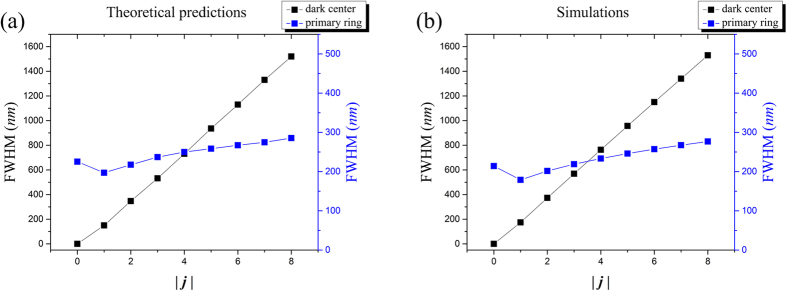
The relationship between FWHM of the near-field radiation and the TC of the plasmonic field. (**a**) Theoretical predictions from the solutions of Bessel functions. (**b**) Simulation results from the 3D finite element method modeling.

From equation (4) it can be seen that the field distribution is described by the Bessel function of the first kind. Thus this simple analytical expression allows us to examine the sizes of both the primary ring and the dark center of the near-field radiation. If the main lobe is a *J*_0_-Bessel function, the size of the dark center is 0 and the full-width-half-maximum (FWHM) of the central peak will be evaluated as the primary ring size. For the other cases where the main lobe is given by higher order Bessel function, both the sizes of the primary ring and the central dark spot will be evaluated by the FWHM of the intensity separation across the primary ring and the dark center, respectively. Figure 3(a) shows the relationship between FWHM of the near-field radiation and the TC of the plasmonic field calculated by the theoretical expression. Clearly the central dark spot expands its size linearly with increasing |*j*|, while the primary ring size increases with the same trend albeit at a much slower rate.

### Numerical modeling with three-dimensional (3D) finite element method

**Figure 4:**
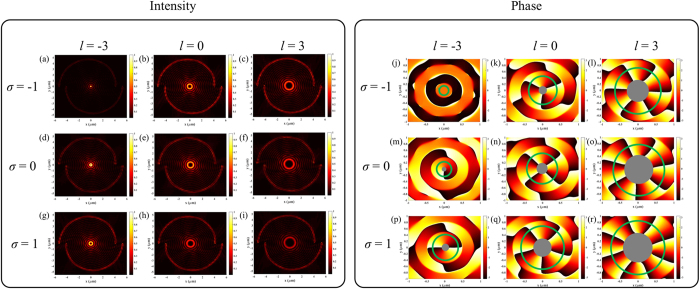
Simulation results for the SBPVL with (*m, n*) = (2, 2) and illumination with different AMs. (**a–i**) Intensity and (**j–r**) phase distributions. The top, middle and bottom rows are corresponding to LHC (*σ* = −1), radial (*σ* = 0) and RHC (*σ* = 1) polarizations, respectively. The TC of illumination varies from –3 to 3 with increment of 3 from the left to the right column in each panel.

The analytical derivations above provide valuable insights into the characteristics of the SPPs vortex synthesized by the SBPVL. However, it is only suitable for a SBPVL that is relatively large compared with *λ*_*spp*_, and the propagation loss of SPPs is ignored. To take all the effects into consideration and validate the analytical predictions, numerical simulation with 3D finite element method model (COMSOL Multiphysics) is performed. The illumination wavelength is chosen to be 633 nm. A SBPVL structure with slot width of 200 nm is etched through a 150 nm gold film (*n* = 0.197 + *i*3.0908) deposited on a glass substrate (*n* = 1.5). The wavelength of the SPPs propagating on the gold/air interface is calculated to be 598.8 nm and *r*_0_ is set to be 4 *μ*m. Simulation results of the near-field (10 nm above the SBPVL surface) intensity and the corresponding *E*_*z*_ phase distributions for SBPVL with (*m, n*) = (2, 2) and illumination with different AMs are shown in Fig. 4. Note that in order to better illustrate the topological charge, the corresponding area of dark center is blocked with a solid circle in the phase map. Good agreements have been obtained between the numerical simulations and analytical predications in terms of the AM conservation. Figure 3(b) shows the numerically calculated FWHMs of the primary rings and dark center, which confirm well with the solution of Bessel functions shown in Fig. 3(a). In summary, these numerical simulations demonstrate that the AM of the illumination play an important role in synthesizing the plasmonic vortex. In addition, the validity of the theoretical expression is proven and it provides a rough but quick estimation of the FWHM of the near-field radiation.

**Figure 5:**
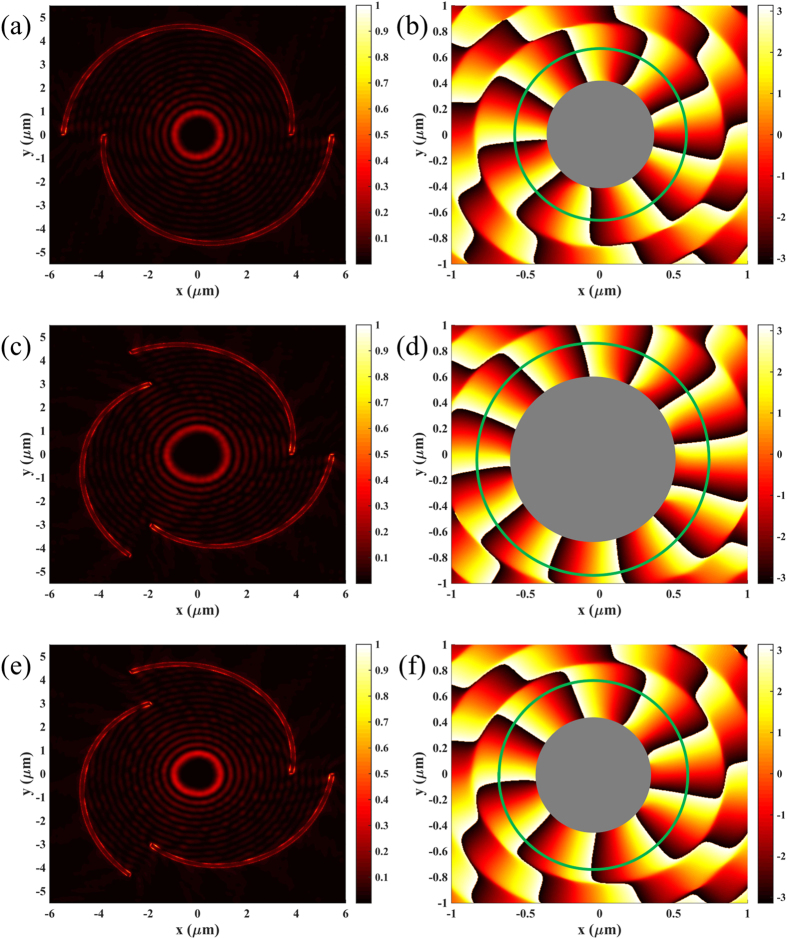
Effect of the geometrical TC of the SBPVL. Intensity and phase distributions for configurations with (**a,b**) (*m, n, σ, l*) = (2, 3, 1, 2), (**c,d**) (*m, n, σ, l*) = (3, 3, 1, 2) and (e, f) (*m, n, σ, l*) = (3, 3, −1, 1).

Considering an incremental length of the slot aperture with orientation angle of *α*, the transmitted field is linearly polarized along *α* direction and carries an additional geometric phase. In this SBPVL structure, in addition to the geometric phase of the SPPs due to a polarization-dependent coupling, a dynamic phase arises as a result of a space-variant path difference, which is induced by the geometrical TC of the SBPVL structure. From the AM selection rule one can see that the geometrical TC of the SBPVL is governed by the product of *m* and *n*, which are the number of blade element and the pitch of blade in the units of *λ*_*spp*_, respectively. To prove this, SBPVL with (*m, n*) = (2, 3) and (3, 3) are numerically studied and the simulation results are summarized in Fig. 5(a–d) respectively, for which the illumination is kept as (*σ, l*) = (1, 2). There are significant differences in the intensity distributions between these two cases (shown in Fig. 5(a,c)), indicating the formation of plasmonic vortex fields with different TCs as shown in Fig. 5(b,d). Note that the asymmetry of the intensity pattern is due to the anisotropic SBPVL structure and propagation loss of SPPs. However, according to the AM selection rule, it is possible to convert different optical complex fields into the same plasmonic field by adjusting the parameters of the SBPVL. As shown in Fig. 5(e), the vortex radii of the configuration with (*m, n, σ, l*) = (3, 3, −1, 1) are the same with that of the case given in Fig. 5(a) with (*m, n, σ, l*) = (2, 3, 1, 2). To this point, the connection between the geometrical TC and the SBPVL structure has been established. Therefore, the TC of the plasmonic vortex field will increase nonlinearly by introducing more blade element to the SBPVL on the premise that the pitch is larger than 1 in the unit of *λ*_*spp*_.

### Effect of the fractional parameters

**Figure 6:**
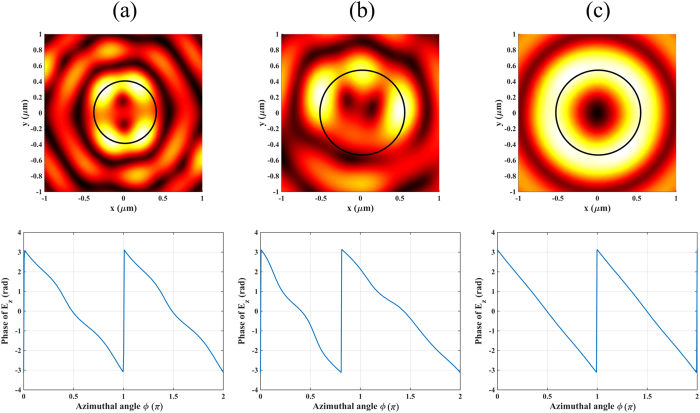
Effect of the fractional parameters. Upper row: Intensity pattern. Bottom row: Phase distribution along the circle indicated in the intensity plot. Configurations with (**a**) (*m, n, σ, l*) = (2, 1.8, 1, −3), (**b**) (*m, n, σ, l*) = (2, 2, 1, −2.6) and (**c**) (*m, n, σ, l*) = (2, 1.8, 1, −2.6).

The aforementioned properties are only considered for the configurations with all the parameters (*m, n, σ, l*) are integers. In practice the non-integer situations exist due to the wavelength mismatching between the illumination and the SBPVL that is designed to work at (*l* becomes non-integer), or the fabrication error of the pitch (*n* becomes non-integer). The top row of Fig. 6(a,b) show the intensity distributions with (*m, n, σ, l*) = (2, 1.8, 1, −3) and (2, 2, 1, −2.6), respectively. The corresponding phase distributions along the circles given in the intensity plots are also presented in the bottom row of Fig. 6(a,b). Noted that when *j* is fractional the number of vortices in the main lobe is rounded to the nearest integer^26^. Besides, it can be seen that the phase varies nonlinearly when *l* and *n* have fractional values, resulting in the symmetry breaking of the intensity patterns. To test whether the AM selection rule of this plasmonic system is still valid for fractional values, configuration with (*m, n, σ, l*) = (2, 1.8, 1, −2.6) is studied in which both *n* and *l* are non-integer however the sum *l* + *σ* + *m* × *n* is an integer. As shown in Fig. 6(c), the phase varies linearly with respect to the azimuthal angles in this case, leading to a SPPs vortex with symmetric intensity pattern and TC of 2, which are the same as that of (*m, n, σ, l*) = (2, 2, −3, 1) illustrated in Fig. 4. Other than *n* and *l, σ* may have fractional values as well, which happens when the polarization of light is elliptical or linear. In these cases, the intensity of the generated SPPs along the slot varies with the position. The non-uniformly distributed SPPs will also give rise to asymmetric intensity pattern.

### Focusing of linearly polarized vortex beam

**Figure 7:**
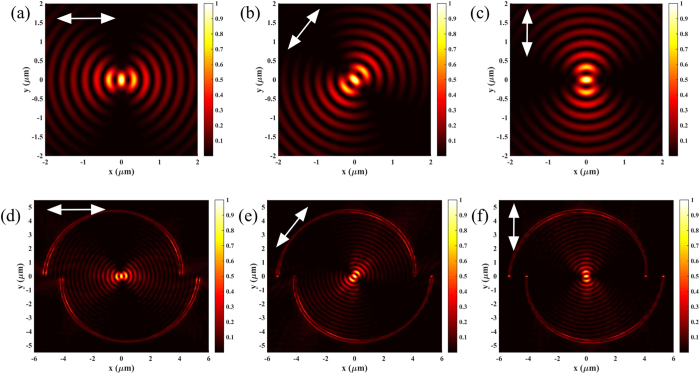
Focusing of linearly polarized vortex beam. (**a–c**) Theoretical calculation and (**d–f**) numerical simulation results of the plasmonic field distributions with (*m, n, l*) = (2, 2, −3) excited by linearly polarized vortex beam. The white arrows indicate the incident illumination with different electric field component angles.

From the above discussions one can find that the key to focus the optical complex field into a highly confined solid spot is to have a plasmonic structure that obtains exactly the negative of the TC of the illumination. However, this requirement is only correct for light with integer *σ* but does not apply to linear polarization. Generally a plasmonic lens with axial symmetry cannot focus a linearly polarized light. Taking the Bull’s eye plasmonic lens as an example, the SPPs waves located at every two opposite points excited by linear polarization have their *E*_*z*_ pointing in the opposed *z* directions, resulting in a inhomogeneous intensity distribution at the center^27^. The asymmetric SBPVL provides an opportunity to achieve a homogeneous focusing spot for linear polarization. When the radius mismatch is 0.5 × *λ*_*spp*_ for every two opposite points, the SPPs waves launched from these two points excited by linear polarization have a relative phase shift of *π*, leading to a constructive interference at the center. Considering a linearly polarized vortex beam normally illuminates the SBPVL, the plasmonic field near the origin can be expressed as:
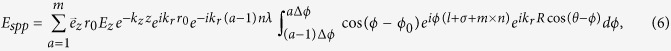
where *ϕ*_0_ denotes the angle of the polarization with respect to the *x* axis, and the cos*ϕ* term is due to the fact that only the electric filed that is locally TM polarized with respect to the slit can be coupled into SPPs. The general requirement to focus a linearly polarized vortex light can be expressed as |*m*×*n*–*l*| = 1. Figure 7 shows the SPPs intensity distribution with (*m, n, l*) = (2, 2, −3) excited by linear polarization from both theoretical predictions and numerical simulations. The white arrow indicates the polarization direction. A solid focusing spot could be observed at the center as expected. Besides, the intensity pattern rotates with the direction of the linear polarization because the SPPs only can be excited along the direction parallel to the incident polarization.

## Discussion

In conclusions, we proposed a SBPVL design that is suitable to convert the optical complex field into tailorable plasmonic vortex. Both theoretical and numerical methods have been adopted to study the overall effect on the characteristics of the synthesized plasmonic vortex field arising from not only the AM of the light, but also the geometrical TC of the SBPVL structure. Besides, it is demonstrated that the geometrical TC arising from the SBPVL with inhomogeneous boundaries is determined by the nonlinear superposition of the pitch and number of blade. The TC of the plasmonic vortex can be found with the AM conservation law (*j* = *σ* + *l* + *m* × *n*), which applies to both fractional and integer parameters. Besides, the sizes of the dark center and the primary ring depend on the solution of the |*j*|-order Bessel function of the first kind, enabling a quick estimation of the FWHMs of the near-field radiation pattern. Moreover, it is demonstrated that by specifically adjusting the structure parameters the SBPVL can also focus the linearly polarized vortex light regardless its polarization directions. This work opens a new avenue to manipulate the plasmonic vortex in subwavelength regime and may find many important applications in optical trapping, optical data storage and other related fields.

## Methods

### Simulation method

The full-wave simulations of the characteristics of the SBPVL are performed using the radio frequency module of the commercial software COMSOL. The device is surrounded by perfectly matched layers, which are used to absorb the scattering optical fields. Both the near-field intensity and phase distributions are calculated by the post-processing available in COMSOL.

### Theoretical derivation of the plasmonic field expression

The electric field at an observation point can be expressed as:
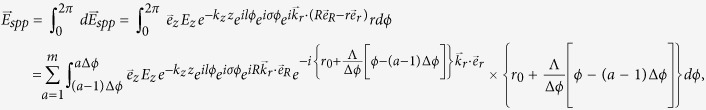
where the SBPVL equation given in equation (1) has been used. Neglecting the propagation loss of the SPP, i.e. Im(*k*_*r*_) ≈ 0, and using Λ = *nλ*_*spp*_, Δ*ϕ* = 2*π*/*m*, 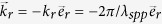, we have:
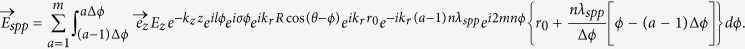


Assuming the structure is large enough with respect to *λ*_*spp*_, its size can be approximated with *r*_0_, so that the equation (8) can be rewritten as:
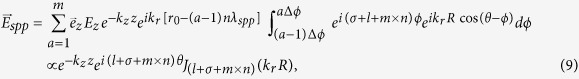
where the integral identify of Bessel function has been used.

## Additional Information

**How to cite this article**: Rui, G. *et al.* Tailoring optical complex field with spiral blade plasmonic vortex lens. *Sci. Rep.* **5**, 13732; doi: 10.1038/srep13732 (2015).
